# Advancing Equity Through Centering Societal Values to Operationalize Racism as a Public Health Crisis: The KKey Values Inequities Model

**DOI:** 10.1089/heq.2023.0113

**Published:** 2023-09-13

**Authors:** Kent D. Key, Jennifer Carrera, Darcy Jones McMaughan, Lisa Lapeyrouse, Roula Hawa, Artina Carter, Sarah Bailey, Vanessa de Danzine, Courtney Blanchard, Jasmine Hall, Nayyirah Shariff, Maji Hailemariam, Jennifer Johnson

**Affiliations:** ^1^ Charles Stewart Mott Department of Public Health, Michigan State University, Flint, Michigan, USA.; ^2^ Department of Sociology, Michigan State University, East Lansing, Michigan, USA.; ^3^ College of Education and Human Sciences School of Community Health Sciences, Department of Counseling and Counseling Psychology, Oklahoma State University, Stillwater, Oklahoma, USA.; ^4^ Department of Public Health and Health Sciences, University of Michigan, Flint, Michigan, USA.; ^5^ School of Behavioural and Social Sciences, Brescia University, London, Ontario, Canada.; ^6^ Flint Community Member, Flint, Michigan, USA.; ^7^ Bridges Into the Future, Flint, Michigan, USA.; ^8^ Community-Based Organization Partners–Brooklyn, Brooklyn, New York, USA.; ^9^ Flint Rising, Flint, Michigan, USA.

**Keywords:** equity, disparities, values, health

## Abstract

**Background:**

The past two decades have been marked by increased efforts to advance equity in various disciplines, including social sciences, public health, environmental health, and medicine. In 2020, a national movement of municipalities declared racism a public health crisis. These efforts have coincided and likely shaped a growing sphere of federal and philanthropic funding for health equity, which frequently calls for practical interventions toward reducing and ultimately eliminating disparities. Disparities in health such as maternal mortality, infant mortality, diabetes, cancer, and stroke have been linked to root causes such as racism. Often, root causes are also linked to disparities in other sectors (i.e., finance/wealth attainment, educational attainment, career attainment, and home ownership). In 2021, in a study published in the New England Journal of Medicine, suggested that racist policies were root causes of U.S. racial health inequities. While racism, sexism, and classism, etc., are characterized as root causes, we posit that there is a deeper driver that has yet to be advanced. This presents a disparity–inequity model that maps disparities and inequities to the societal value system, not root causes.

**Methods:**

The KKey Values Inequities Disparities Model described in this article combines a case study of the Flint Water Crisis to explore the historic impact of human devaluation and its role in systemic racism and classism, which ultimately creates and exacerbates inequities that produce disparities in communities. The model integrates the value system and its contribution to societal causes (formerly known as root causes).

**Conclusions:**

A broadly defined values–inequities–disparities model will allow researchers, practitioners, decision makers, lawmakers, and community members to (1) assess the core root of inequities and disparities; (2) identify solutions in the human value domain; (3) design appropriate course corrective programming, interventions, processes, and procedures; and (4) create actions to integrate new systemic procedures and practices in our laws and governance to advance equity.

## Introduction

### Case

Flint, Michigan, a majority African American city, was put under the control of an emergency manager (EM) appointed by the State Governor because of Flint's financial distress. EMs usurp the power of local elected officials, resulting in a loss of local democracy that yields no power in the decision-making process.^1,2^ The EM decided to switch Flint's water source from the Detroit Water Authority to the Flint River. In addition, a decision was made to not add anticorrosion control (for less than $150 a day) to the water. Immediately following the water switch, residents began to complain of foul odor, taste, and various health conditions.

A few months later, General Motors complained that the water was damaging their parts at their Flint Plants. The State of Michigan allowed General Motors to return to using the Detroit water, while forcing residents to remain on the Flint River water. On February 17, 2017, the Michigan Civil Rights Commission, after gathering evidence and facts and holding three hearings, concluded that the Flint Water Crisis was an act of systemic racism.^3^

Racism has been declared a public health crisis in the United States by over 240 municipalities.^4^ These declarations represent an important step in helping individuals and communities amplify the critical impact racism has on the health and well-being of populations of color. Importantly, these declarations also provide a new opportunity to conceptualize racism and particularly systemic racism's impact on population health.

Inequities experienced by people of color have been attributed to personal shortcomings and unhealthy cultural preferences and practices^5^; a more complex understanding of racial/ethnic health inequities points to long-standing inequities in health resources (i.e., health care, income, housing, and education) that result from racism embedded within our social systems, policies, and practices.

Declaring racism a public health crisis suggests a public health response to racism. Public health lends itself to this mass movement. There is literature supporting the view that effective public health responses to racism reduce and ultimately eliminate systemic and structural inequities that contribute to health disparities (HD). For the past few decades, scholars have explored the impact of racism as a “root cause” of HD.^6,7^ More recently, Bailey et al. (2021) examined how racist policies serve as a root cause of racial health inequities and how structural racism works in the United States.^8^

The goal of this article is threefold: (1) to respond to two of the four outlined key areas identified by Bailey et al.^8^; (2) to propose a new model, the KKey Values Inequities Model (KVIM), to address key areas 3 and 4 in the study by Bailey et al.^8^; and (3) to present this model as a guide to advance equity by centering the societal value system. The KVIM challenges the current perception of root causes (racism) and posits that the real root cause of disparities and inequities is the societal value system, which in and of itself is the root cause of racism and all other “isms.”

The KVIM integrates value system models with a disparity/inequity model. It suggests that root causes stem from the value system. We posit that if you start in the domain of the societal value system and correct that domain, it will self correct in all other domains, which will advance equity, eliminate inequities, and thus reduce HD. Furthermore, we argue the need to clarify the term “root cause” and offer a different perspective.

While we understand that no one tool, model, or framework will eliminate disparities, this model can serve as one of many tools communities can use to operationalize racism as a public health crisis by focusing on societal values as a starting point to address HD.

## Methods

As we developed our proposed model, we explored a plethora of literature, ranging from research studies, systematic reviews, and existing frameworks, models, and theories, to identify the domains needed in a comprehensive model to address inequities and disparities and to further explore the impact racism has on the overall health and well-being of people.

### Literature review/background

The New England Journal of Medicine released a publication in 2021, sharing how structural racism works and positing that racist policies are the root cause of U.S. racial health inequities.^8^

Bailey et al.^8^ highlight examples of racist policies such as redlining and racialized residential segregation that impact health. In addition, the article highlights (1) racist policies and practices in the justice system, specifically around policing and prison demographics, which disproportionately impact communities of color; and (2) unequal health care, exploring the historical roots of scientific racism and eugenics movements in modern-day studies of inequitable treatment experienced by people of color.

Equally important, the article suggests that U.S. institutions must engage in four key areas to move beyond individual education and personal insight to achieve transformative policy and social change. Those key areas are (1) embracing the intellectual project of documenting the health impact of racism; (2) improving and ensuring the availability of data that include race and ethnicity and improve the measurement of structural racism; (3) having medical and public health communities turn the lens on themselves both as individuals and as institutions; and (4) acknowledging that structural racism has been most successfully challenged by mass social movements.

The aforementioned points provide a road map for dismantling structural racism. For the purpose of this article, we will concentrate on key areas 3 and 4.

A wealth of research exists exploring the *relationship between racism and poor physical and mental health* outcomes across the life course.^9–11^ Racial/ethnic inequities in maternal mortality,^12^ infant mortality,^13,14^ diabetes,^15^ stroke,^16^ cardiovascular disease,^17^ and hypertension^18^ have been linked to racism. Most recently, racial/ethnic disparities as a result of COVID-19 (cases, hospitalizations, and deaths) were trending heavily in 2020–2021,^19–21^ underscoring the ongoing impact of racism on health and well-being during a public health pandemic.

In recent years, emphasis has been put on the *structural and systemic* nature of racism and the role that it plays in systems such as health,^22,23^ employment,^24^ education,^25^ socioeconomic status,^26^ housing,^27^ transportation,^28^ the built environment (zip codes),^29^ and food access.^30^ These systems (not exhaustive) are in alignment with the social determinants of health (SDOH) framework—a public health framework that aims to identify and understand how conditions in the places where people live, learn, work, and play affect a wide range of health risks and outcomes.^31,32^

This approach enables the field of public health to look at the social interactions of the societal environment (institutions, laws, policies, and systems, etc.…) and how they affect or influence the overall health and well-being of people, not only in clinical care but also socially. The historic and consistent deaths of people of color when engaging with law enforcement is an example of racial disparities leading to people of color dying at greater rates when engaging with law enforcement.^33^

In addition, a continuously growing body of *epidemiological evidence* has documented the negative impact of racism on health,^34^ with scant progress made toward eradicating these impacts.^35^ Scientific reviews have shown that racism impacts the physical and mental health of children and youth of color.^36^ An earlier literature review examining 121 studies found statistically significant associations between racial discrimination and mental health in youth of color.^37^

Reviewing data from studies conducted between 1983 and 2013 and reported in 333 articles, Paradies et al. concluded that racism was associated with poorer mental and physical health among adults.^38^

#### Interdisciplinary problem

The issue of racial inequities and racism is documented across disciplines, including health and medicine,^9,39^ sociology and economics,^40^ social work,^41^ and education.^42^ This problem is systemic and a result of historical and contemporary oppression.^43^ Racism is a structured system that interacts with and shapes social institutions.^23^

As with all systems, racism has created systems with their subcomponents. These systems and subsystems are dynamic, interdependent, and reinforce each other, creating and sustaining reciprocal causality of racial inequities across the various systems and sectors in society.^23^ To address a system-structured problem, we must provide a system-structured solution. The field of public health provides system-level approaches, frameworks, and models that can aid in this work.

#### Dehumanization/devaluing

We posit that the origin of the systemic nature of racism and racial inequities begins with the concept of dehumanization, ascribing value. To advance equity and eliminate disparities, we saw a need for a model that incorporates the underlying values that drive inequities. Antonio Gramsci argued that structural change was impeded by the entrenched hegemonic ideology of the dominant cultural values.^44^

The work of addressing system-structured problems requires an approach that addresses the entrenched ideology that supports inequitable public health outcomes. Piecemeal approaches that attempt to address decontextualized oppressions allow for slippage to other interlinked oppressions as fundamentally the hegemonic ideological basis for inequity is not *just* racism or classism but also at its core a willingness to accept inferior conditions for some groups because they are not recognized as being fully deserving of the same rights and privileges.

Dehumanization underlies a system that allows for all -isms to exist. Transforming the structure that allows for socioeconomic and race-based inequalities to persist then requires an approach that directly addresses and disrupts the mechanisms that rationalize and perpetuate processes of dehumanization.^45^

**FIG. 1.**
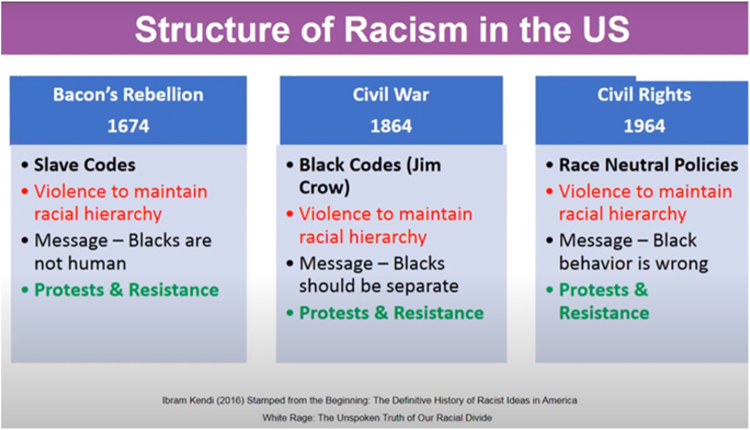
Structure of racism in the United States.

Dehumanization in the United States began with the founding document, the United States Constitution. The original preamended document ascribes a value to “slaves” (those of African descent) as three-fifths of a human, less than human. It further declares slaves were not human, but “chattel” property (U.S. Constitution).^46,47^ It was clear dehumanization, ascribing a lesser than human value to a human being. This negative devaluing of African Americans progressed and morphed over time from the constitution to racist laws and policies. Kendi shows that progression from the slave codes to civil rights (Fig. 1).^48^

Dehumanization in the United States went beyond racism toward enslaved Africans and Africans born in America. Native Americans, Latin Americans, Mexican immigrants, and Muslims^49^ and other immigrants now considered white (Irish and Jews) were also racially discriminated against.^50,51^ The women's liberation movement in America was a response to the dehumanization of women compared with men.^52^

**FIG. 2.**
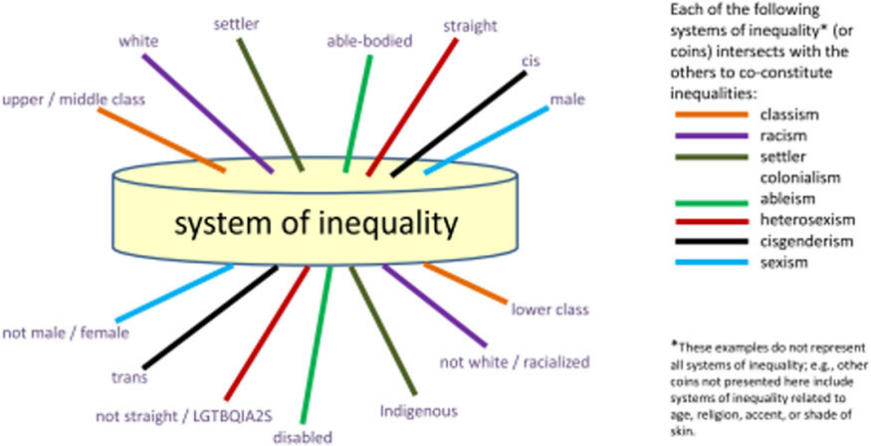
The intersecting nature of the coin: patterns of advantage and disadvantage. *Source:* Nixon.^54^

More recently, the Lesbian, Gay, Bisexual, Transgender, Queer, Intersex, Asexual (LGBTQIA) community has experienced dehumanization and lesser value in their treatment and efforts to achieve equality in the workplace, education, gay marriage, and other initiatives.^53^ Being ascribed a lessor or greater value as a human is an indicator of the advantage/privilege or disadvantage/lack of privilege one may experience. One framework that captures and summarizes dehumanization in terms of advantaged and disadvantaged humans is shared by Nixon in Figure 2.^54^

#### Value system models

Value system models have expanded and evolved over time and have been used across multiple disciplines. In the behavioral sciences, value system models draw from the theory of planned behavior. Used to predict and understand human behavior, this theory is an extension of the theory of reasoned action. The theory of planned behavior posits that behavioral achievement is dependent on intention and behavioral control, placing self-efficacy belief or perceived control within the framework of beliefs, attitudes, intentions, and behaviors.^55,56^

In the environmental science field, values directly impact behavioral commitment (values–beliefs–norms–behavior commitment). In social psychology, the value–belief–norm theory model posits values as the genesis of behavior. In this model, values directly impact beliefs, beliefs directly impact personal norms, and personal norms directly impact behaviors.^57^ This model and variations of it have been used across various social movements.^58^

#### Root causes and SDOH

**FIG. 3.**
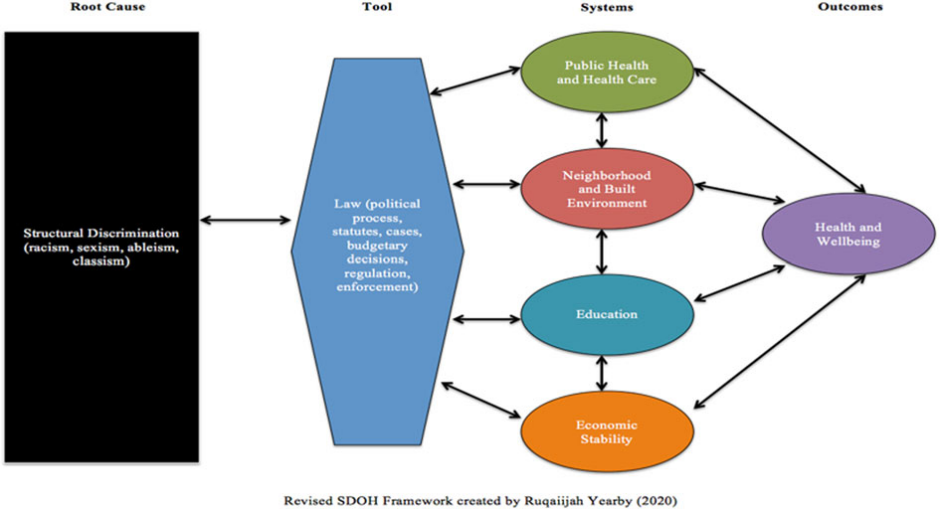
Revised social determinants of health framework created by Yearby.^7^

Pre-existing public health models addressing SDOH and HD have centered on root causes. Building upon the Healthy People 2020 SDOH framework,^31^ Yearby posits that root causes and the “law” (political processes, statutes, budgetary decisions, regulations, and enforcement) have a bidirectional relationship. In addition, law directly impacts systems (that public health classifies as SDOH) and those systems directly impact health and well-being (Fig. 3).^7^

**FIG. 4.**
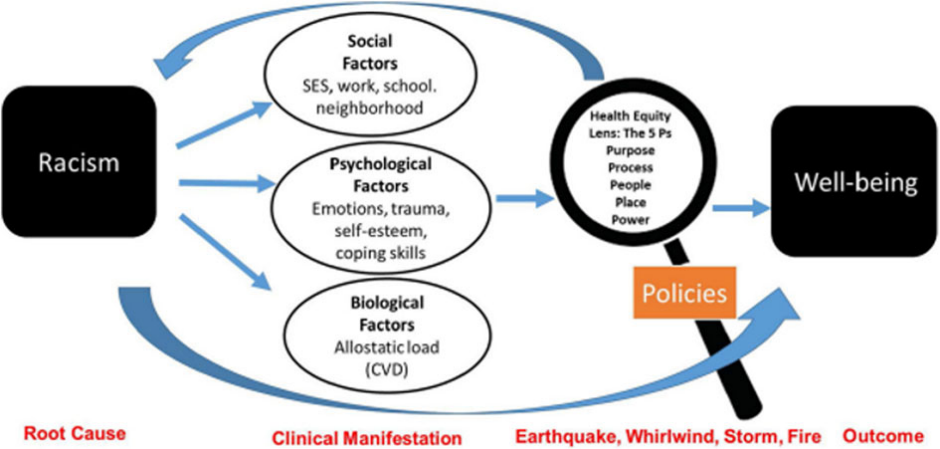
Biopsychosocial model. *Source:* Sanders and Fiscella.^59^ CVD, cardiovascular disease; SES, socioeconomic status.

In this depiction, researchers identify the law as a tool of structural racism influencing systems across all institutions and sectors.^7^ A similar model from the field of psychiatry expands on the impact of racism and suggests that it is the root cause impacting clinical manifestations (social, psychological, and biological factors), which if intercepted by policy efforts that center equity, will result in well-being, as shown in Figure 4.^59^

### The KVIM

Building upon these previous models and theories, we propose a public health model that integrates SDOH models with value system models. Thus, our goal is to use this model to center efforts addressing racism as a public health crisis around a “societal value system” conversation as the targeted domain to begin creating solutions. This model responds to points 3 and 4 in the Bailey et al. (2021) New England Journal of Medicine article^8^: “3) medical and public health communities need to turn the lens on themselves both as individuals and as institutions; and 4) acknowledge that structural racism has been most successfully challenged by mass social movements.”

**FIG. 5.**
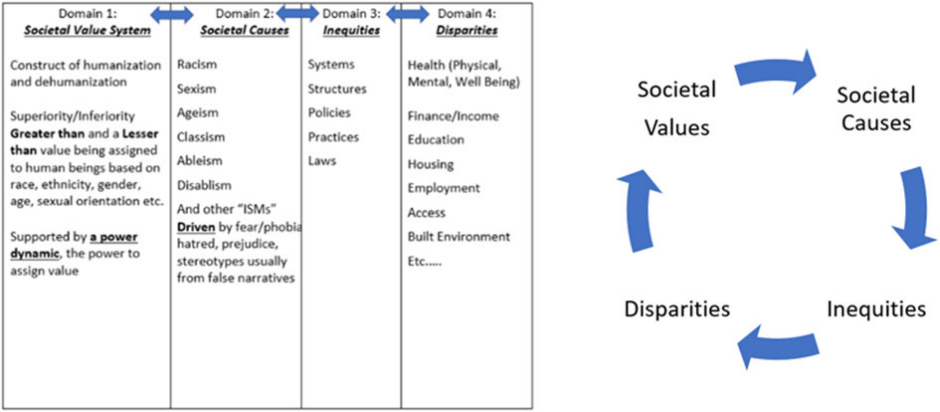
KKey Values Inequities Model.

This would allow researchers, practitioners, policy makers, and community members to (1) reassess their values, (2) explore how their values drive root causes, (3) explore the impact that root cause has on inequities, and (4) provide actionable and identifiable strategies and leverage points to reduce and ultimately eliminate disparities. The aforementioned value system models support the notion that behaviors and outcomes are the result of values on a personal level. We posit through our proposed KVIM (Fig. 5) that this same pattern also impacts societal and system levels.

The KVIM was developed, in large part, through direct observations of the need of community, academics, local government, and activists to (1) operationalize the declaration that racism is a public health crisis, (2) address the value system and its impact on disparities, and (3) create a public health model that integrates both value system and SDOH domains.

We begin by adding a value system domain to a root cause model. **Domain 1: Societal Value System.** We define the value system (as a societal-level value system) with a focus on how our society values the humanity of all people. In this domain, we identify the construct of humanization and dehumanization. We posit that our current societal value system has been subjugated to a duality of superiority and inferiority. In this domain, society places unequal values on human beings (grouped by race, ethnicity, gender, age, and sexual orientation, etc.). Within this domain, a power dynamic is established and preference and privilege are established.

**Domain 2: Societal Causes.** As a result of the value system domain, societal causes manifest through belief systems and ideologies. We also argue that the traditional classification of root causes (racism, sexism, ageism, classism, and ableism, etc.) is not the root, societal values are the root. Thus, we identify these as societal causes. These societal causes (mislabeled as root causes) have been researched, studied, identified, and linked to disparities in scholarly literature for over the last three decades or more.

**Domain 3: Inequities.** Societal causes (ideologies and beliefs) are manifested through the people who (1) run our systems, (2) enforce policies and regulations, (3) create and enforce laws, (4) guide our practice, and (5) govern our institutions, thus creating systemic inequities (inequity domain).

**Domain 4: Disparities.** As a result of the inequities that are fueled by a societal value system and sustained by societal causes (formerly known as root causes), we (as a society) produce disparities in health, economics, education, housing, transportation, employment, and the built environment, etc., which align with domains of the SDOH in public health. Conceptually, we agree with Yearby that law is a tool that produces disparities; however, we posit that law is one of many inequities (tools) that produce disparities manifested within the domains of the SDOH.^7^

More importantly, we stress the historical misclassification and misidentification of root causes, contributing to failed attempts and interventions in eliminating HD. Focusing on racism as the root cause of disparities is in error. We must focus on the societal value system and intervene at that level if we want to dismantle the societal causes (formerly known as root causes) that create the inequities that produce disparities.

In addition, we argue that the KVIM is cyclical in nature. Apparent in the KVIM, societal values precede societal causes, societal causes precede inequities, and inequities produce the disparities; however, we argue that disparities manifesting in certain subpopulations may reinforce how the dominant society views and values those human beings. Thus, a reinforcement loop is created.

Given the declaration that racism is a public health crisis, the KVIM is poised to address disparities and SDOH by shifting the focus to societal values. This approach could offset the immediate tension and possible denial of societal causes (root causes). From the literature, we know that when engaging in conversations concerning root causes, those who benefit from them tend to feel guilt and/or go into denial, which prevents productive discourse and solution generation.

As shown in Figure 2 in the two sides of the coin of the privilege model, root causes produce two groups of human beings: the privileged (superior) and the oppressed (inferior). Figure 2 goes further to give contemporary examples of those classifications of human beings. Figure 2 is reflected in KVIM domains (value systems and root causes). Note that this model is not just a racism-specific model but it can also be used to address multiple root causes (classism, sexism, colonialism, and ableism, etc.). For the purposes of this article, we focus on racism given that it has been declared a public health crisis.

We emphasize the fact that this model shifts efforts and conversations toward values and not root causes as there is a root to the root causes. Starting conversations at root causes creates tensions between those traditionally privileged and those traditionally oppressed. The feeling of guilt, which leads to feelings of distress among people who are privileged (receiving unearned benefits), can lead to distancing from the issue, denial, and intellectual paralysis.^54^

Those traditionally privileged feel the need to avoid and/or justify that they have earned the privilege. Furthermore, Nixon posits “Discussions of privilege can lead to faulty assumptions of innocence and counterproductive attention to guilt.”^54^ This creates even wider barriers to creating solutions, especially if those oppressed are told to just work harder or that they are not working hard enough according to the myth of meritocracy.^60^

By focusing on societal values, our model suggest that conversations about racism should not include blaming others for the historical past of our forefathers, but rather our conversations should start with discussing how we value each other (present day), how we value human beings (who may be different from oneself), and if we value them as equal to ourselves. This approach aids in finding shared values regarding humanity.

This is critical if we are to discuss the historical societal value system and the need to correct it to address societal causes, inequities, and ultimately disparities.

## Discussion

In our case study, the value of human lives was overlooked for the sake of fiscal belt-tightening. If the water in Flint, Michigan, was toxic enough to destroy automobile parts, was it not toxic enough to damage human tissues and organs and cause other health issues? This case is a classic example of the devaluing and dehumanization of human beings. The message to Flint residents was clear: the powers that be valued automobile manufacturing and potential profits from it over the lives of people (adults and children alike).

The conclusion of systemic racism by the Michigan Civil Rights Commission further underscored the need to address the inequities and disparities caused by racism and other “isms.” Recognizing and centering the societal human value system were not a priority in this case, as suggested in the KVIM.

## Future Directions and Implications on Public Health and Advancing Equity

Declaration of racism as a public health crisis allows us to look at racism from a system level and to go beyond individual experiences. It is a triumph to get the resolution drafted and adopted by local municipalities. However, the questions “So what?” and “Now what?” must be answered. This model can assist in identifying next steps after passing the resolution.

This KVIM helps us address disparities using the domains (systems) of the SDOH, which have contributed to the racial and ethnic health (physical and mental) disparities. In addition, this model can be used across all disparity-focused fields. Finally, this model was created and vetted by both academic and community partners and revised with input from the broader community.

### Public health

The KVIM has implications for operationalizing racism as a public health crisis declaration. With ∼250 declarations in the United States, there is a need to develop a tool kit of strategies, plans, and other resources for those municipalities. There is no one-size-fits-all approach to operationalizing the resolutions, therefore creation of a tool kit with a wide range of resources is needed.

More importantly, the KVIM is a public health model derived from public health models addressing HD and SDOH with the added societal value system domain.

### Community

The KVIM can be used by local community advocates and activists focused on advancement of equity and elimination of disparities. It provides a frame by which community conversations, initiatives, healing circles, and other efforts can center conversations on human value and create solutions to address racism and HD. Equally important, it can serve as a caveat to systems interventions and approaches addressing the SDOH.

### Policy

The KVIM informs the types of policy training interventions that policy makers should receive to address disparities in their local communities. Furthermore, the model depicts how inequities are sustained by root causes and values, which manifest in disparities. Furthermore, those most impacted by racism are often excluded from the decision-making processes.

Thus, it is important that (1) policy makers are educated, involved, and informed about the impact of racism as a public health crisis and the role the value system plays in the equation and (2) effective community engagement spaces are created so that policy makers can hear firsthand the impacts of racism on their constituents and cocreate culturally appropriate solutions with them.

### Creating a culture of health

This model underscores the need to center equity in addressing disparities in the United States. Our systems, laws, practices, policies, and procedures must be transformed and reconstructed. Racial equity work must be infused with health equity work if we are to eliminate HD.

## Conclusions

We hope this model serves as a guide for those seeking to operationalize declarations of racism as a public health crisis. In addition, this model may serve as a guide for researchers, community members, and policy makers who are looking for tools to reduce and ultimately eliminate disparities. We hope to underscore the importance of the role that the societal value system plays in production of disparities.

To the extent possible, this information can be shared with health-focused community organizations to enhance their understanding of inequities and disparities. We also anticipate that the KVIM will be adopted by schools of public health and other social science departments to effectively provide models to address disparities.

We further hope to increase the confidence of antiracism activists, researchers, and policy makers in their approach to address racism as a public health crisis.

## Abbreviations Used

EM: emergency manager
HD: health disparities
KVIM: KKey Values Inequities Model
SDOH: social determinants of health
SES: socioeconomic status
